# Analyzing the spatio-temporal relationship between dengue vector larval density and land-use using factor analysis and spatial ring mapping

**DOI:** 10.1186/1471-2458-12-853

**Published:** 2012-10-09

**Authors:** Muhammad Shahzad Sarfraz, Nitin K Tripathi, Taravudh Tipdecho, Thawisak Thongbu, Pornsuk Kerdthong, Marc Souris

**Affiliations:** 1 Remote Sensing and GIS, School of Engineering and Technology, Asian Institute of Technology (AIT), P.O. Box 4, Pathumthani 12120, Klong Luang, Thailand; 2 The Office of Disease Prevention and Control, Bureau of Vector Borne Disease (9th), Ministry of Public Health, Wat bot road, Muang Phitsanulok 65000, Thailand; 3, Institut de Recherche pour le Développement (IRD), UMR 190, Marseille, France

## Abstract

**Background:**

Dengue, a mosquito-borne febrile viral disease, is found in tropical and sub-tropical regions and is now extending its range to temperate regions. The spread of the dengue viruses mainly depends on vector population (*Aedes aegypti* and *Aedes albopictus*), which is influenced by changing climatic conditions and various land-use/land-cover types. Spatial display of the relationship between dengue vector density and land-cover types is required to describe a near-future viral outbreak scenario. This study is aimed at exploring how land-cover types are linked to the behavior of dengue-transmitting mosquitoes.

**Methods:**

Surveys were conducted in 92 villages of Phitsanulok Province Thailand. The sampling was conducted on three separate occasions in the months of March, May and July. Dengue indices, i.e. container index (C.I.), house index (H.I.) and Breteau index (B.I.) were used to map habitats conducible to dengue vector growth. Spatial epidemiological analysis using Bivariate Pearson’s correlation was conducted to evaluate the level of interdependence between larval density and land-use types. Factor analysis using principal component analysis (PCA) with varimax rotation was performed to ascertain the variance among land-use types. Furthermore, spatial ring method was used as to visualize spatially referenced, multivariate and temporal data in single information graphic.

**Results:**

Results of dengue indices showed that the settlements around gasoline stations/workshops, in the vicinity of marsh/swamp and rice paddy appeared to be favorable habitat for dengue vector propagation at highly significant and positive correlation (p = 0.001) in the month of May. Settlements around the institutional areas were highly significant and positively correlated (p = 0.01) with H.I. in the month of March. Moreover, dengue indices in the month of March showed a significant and positive correlation (p <= 0.05) with deciduous forest. The H.I. of people living around horticulture land were significantly and positively correlated (p = 0.05) during the month of May, and perennial vegetation showed a highly significant and positive correlation (p = 0.001) in the month of March with C.I. and significant and positive correlation (p <= 0.05) with B.I., respectively.

**Conclusions:**

The study concluded that gasoline stations/workshops, rice paddy, marsh/swamp and deciduous forests played highly significant role in dengue vector growth. Thus, the spatio-temporal relationships of dengue vector larval density and land-use types may help to predict favorable dengue habitat, and thereby enables public healthcare managers to take precautionary measures to prevent impending dengue outbreak.

## Background

Dengue is a vector-borne disease, estimated to affect millions of individuals each year in tropical and subtropical areas, and it is reemerging in areas that have been disease-free for relatively long periods of time. This expansion is creating new opportunities for viruses to propagate in new areas and is acting as a common cause of epidemics in what were *Aedes (Ae.)* free countries [1], and is associated with numerous problems at local and global scales. Dengue is endemic in tropical and subtropical regions as the viruses that cause it are transmitted through the bite of an already infected mosquito. Epidemics of this mosquito-borne illness are rising worldwide due to increased international travel and unplanned urbanization combined with lack of effective mosquito control measures in tropical developing countries [2]. In recent years, transmission of these viruses has increased predominantly in urban and semi-urban areas, and is a major international public health concern owing to its hold over approximately half of the world’s population. According to estimates by the World Health Organization (WHO), there could be as many as 50 to 100 million dengue infections worldwide each year [3]. In the absence of an effective drug or vaccine, the only strategic options to control dengue are case management to prevent death and control of vectors to reduce viral transmission [4]. The appearance of numerous infectious diseases is strongly facilitated by environmental factors, such as climate or land-use change [5]. The spatial distribution of vector-borne diseases are typically determined by the geographical extent of the vector or reservoir hosts and their habitat priorities [6]. During immature stages, vector species differ in their priorities for habitat. Therefore, change in land-use permits the colonization of new habitats as well as the expansion or reduction of the range of the vector. This may also modify the composition of the mosquito vector community [7]. Agriculture is the largest driver for land-use change across the earth. Croplands and pastures together have become one of the largest terrestrial biomes on the planet, rivaling forest, and cover in extent of 40% of the land surface area [8,9]. Land-use/land-cover patterns and socio-cultural practices have a strong relation with dengue incidence [10]. Vegetation and land-cover characteristics can influence the risk of contracting dengue as they modify mosquito population. Land-use change is a major constituent of global environmental change that can potentially affect human health in relation to mosquito-borne diseases by influencing the mosquito’s habitat. The distribution and abundance of vectors concomitantly mediates human-mosquito interactions including biting rate [11].

Vector population estimates have been carried out by many researchers using dengue indices, i.e. house index (H.I.), container index (C.I.) and Breteau index (B.I.), as indicators that can be adopted to compare their individual relationships with each of the habitat parameters [4,12]. The relationship between number of dengue virus infections (cases) and climatic factors has been reported by many researchers using several indices, and it has been concluded that climatic conditions have a strong influence on dengue infection [13-15]. However, drawbacks associated with the use of dengue case data include the occurrence of asymptomatic infections which cannot be used to support a real-time indication of a forthcoming disease scenario, as can be predicted using larval density data [16]. The dengue vector species mainly contributing to dengue outbreak include *Ae. aegypti* (L) and *Ae. albopictus* (L) [13]. Of both species, female mosquito mostly likes to feed on human blood, whereas male mosquito prefers nectar feeding [17]. The former does not fly far and the majority of the population remains within a 200 meter distance from the site of their emergence [3]. However, this type of mosquito uses a wide range of confined larval habitats, both natural and man-made. On the other hand, larvae of the later species have been found not only in natural containers, i.e. tree holes, plant axils, cut bamboo stumps and opened coconuts, but also in outdoor artificial containers, i.e. rubber tyres, water storage barrels, glass and plastic bottles, and jars located up to one-kilometer diameter [15,18].

Spatial epidemiology is the study of spatial variation in disease risk or incidence. Several ecological processes can result in strong spatial patterns of such risk or incidence, e.g. pathogen dispersal might be highly localized, vectors or reservoirs for pathogens might be spatially restricted, or susceptible hosts might be clumped [19]. In addition to the reported relationship between climatic conditions and dengue infection [13,15], understanding the contribution of land-use type to dengue vector breeding ability is directly required to unveil the underlying scenario comprehensively. A few previous studies have emphasized the analysis of land-use and land-cover types due to their relevance with mosquito breeding habitat and people’s living style. It was pointed out by many researchers that the land-cover can be an important risk determinant for dengue infection [20,21], therefore it demands a comprehensive analysis of surrounding landscape and its possible relationship with the dynamics of mosquito population for subsequent dengue incidence.

The present study was conducted to predict the suitable potential habitat for dengue-transmitting mosquitoes, integrating dengue indices and land-use types using spatial epidemiology and statistical approaches. Moreover, the extent to which land-use types influence dengue-transmitting mosquito growth was also investigated.

## Methods

### Study area

**Figure 1:**
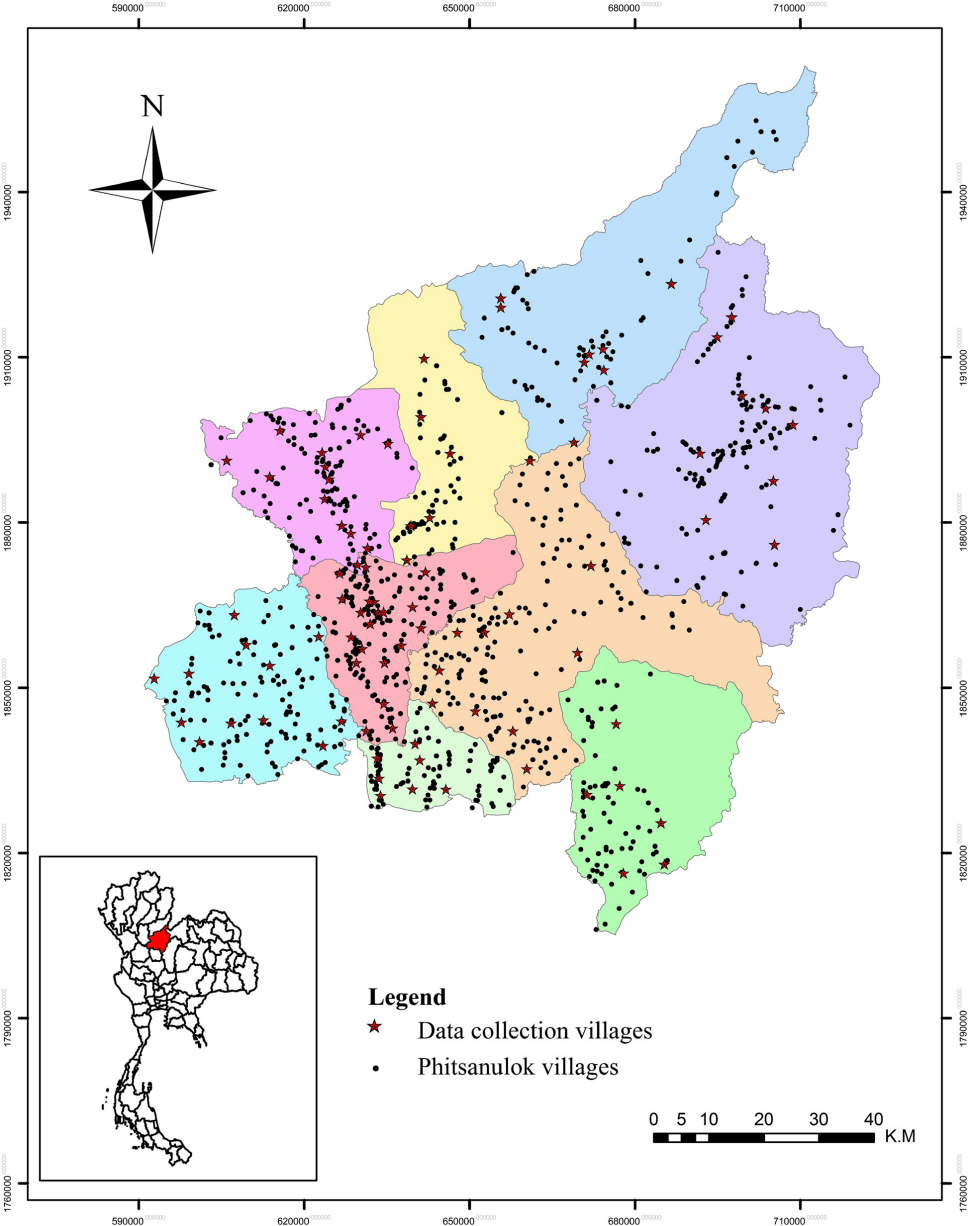
**Phitsanulok Province with district boundaries (study area).** Inset box shows location of Phitsanulok Province (red) in Thailand. Red stars indicate the 92 villages which were sampled three times (i.e. March, May and July) during 2009. Dots indicate all the villages in Phitsanulok Province. Total area of Phitsanulok is approximately 10,816 km^2^.

**Figure 2:**
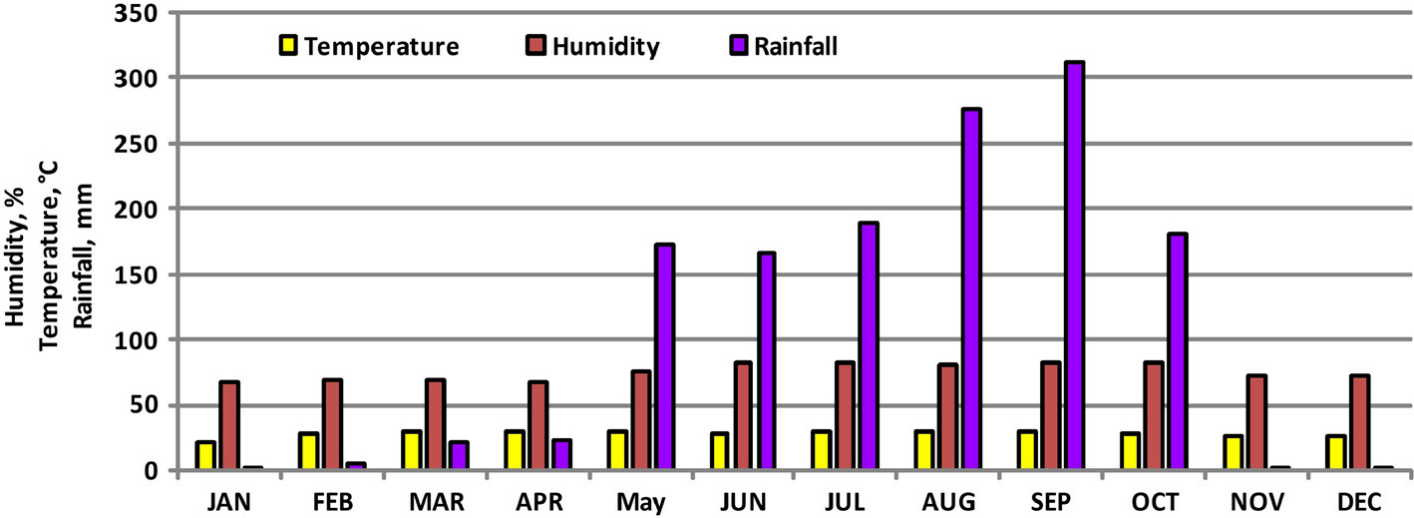
**Total number of patients suffering from various diseases and dengue infection between 2006 and 2010.** Number of suspected dengue cases (cases with clinical symptoms) reported and the total number of patients. Figure shows that dengue cases reported each year vary in the study area.

Phitsanulok is a province of Thailand, located in its lower northern region (16.78 N, 100.20 E), 377 km from Bangkok (Figure 1). The province has a population density of 77.99 (people km-2) and has nine amphoes (districts), ninety-three tambons (sub-districts) and more than one thousand muban (villages) [22]. Villages are considered the smallest unit under Thailand’s public health system. The study area has diverse land-use and land-cover types, e.g. urban, field crops, orchards, mountains, river, waterfalls, swamps, forests, grasslands, caves, reservoirs and an extensive network of canals. The major land-use classes in the study area are urban and crop land, as most people have adopted farming as an occupation. A major outbreak of dengue was witnessed during 2008 when 1385 patients were admitted to hospitals (Figure 2). It was declared the worst outbreak in the history of Phitsanulok Province. The outburst prompted Phitsanulok’s government agencies to study the behavior and pattern of the disease, and also to ascertain possible causes contributing to favorable habitats for its spread. This was done in order to cope with and prevent future outbreaks of such magnitude at the national level, considering Phitsanulok Province on a priority basis.

### Climatic conditions

**Figure 3:**
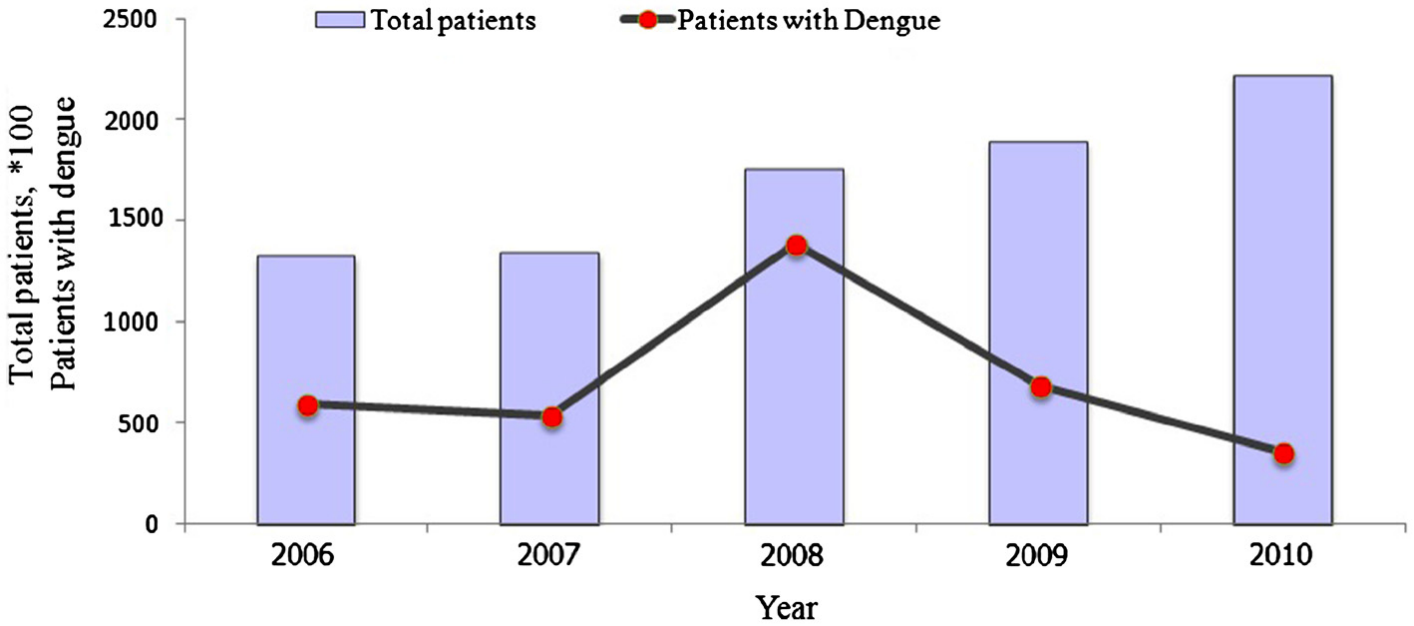
**Changes in temperature, rainfall and humidity from January to December, 2009.** The X-axis shows the monthly average climatic variation, while the Y-axis shows climatic factor ranges such as temperature, humidity and rainfall. Humidity and temperature remained the same throughout the year but rainfall varied. Normally in Phitsanulok the rainy season starts from April and ends in October.

The climate of the province is hot tropical with an annual rainfall of approximately 1800 mm. However, at higher altitudes the weather is cool with a maximum temperature of around 25.0°C and at lower altitudes it is around 33.0°C. During the study period (2009), the mean temperature, humidity and rainfall recorded were 28.0°C, 75.0% and 112.0 mm, respectively (Figure 3).

### Health facilities

The province has good numbers of registered medical treatment units, i.e. 145 health centers and 270 clinics, for its residents [22]. Still there are constant health issues faced by the citizens in the province, with a dramatic increase in the rate of annual in-patients suffering from various kinds of diseases. Dengue fever (D.F.) and dengue hemorrhagic fever (D.H.F.) are among the top five leading diseases in the province.

### Data sources

Data of dengue-transmitting mosquitoes, i.e. house index (H.I.), container index (C.I.) and Breteau index (B.I.), were collected according to WHO standards by the Ministry of Public Health (MOPH), Division-9th, Phitsanulok, Thailand for the year 2009.

Dengue indices were calculated using the following formulas:

$$H.I.=\frac{\mathrm{Number}\;\mathrm{of}\;\mathrm{infested}\;\mathrm{houses}}{\mathrm{Number}\;\mathrm{of}\;\mathrm{inspected}\;\mathrm{houses}}\times 100$$

$$C.I.=\frac{\mathrm{Number}\;\mathrm{of}\;\mathrm{infested}\;\mathrm{containers}}{\mathrm{Number}\;\mathrm{of}\;\mathrm{inspected}\;\mathrm{containers}}\times 100$$

$$B.I.=\frac{\mathrm{Number}\;\mathrm{of}\;\mathrm{infested}\;\mathrm{containers}}{\mathrm{Number}\;\mathrm{of}\;\mathrm{inspected}\;\mathrm{houses}}\times 100$$

Information about land-use and land-cover was obtained from the Land Development Office, Phitsanulok. Data for the administrative boundaries was acquired from the Royal Thai Survey Department of Thailand.

### Data sampling and conversion to GIS

**Figure 4:**
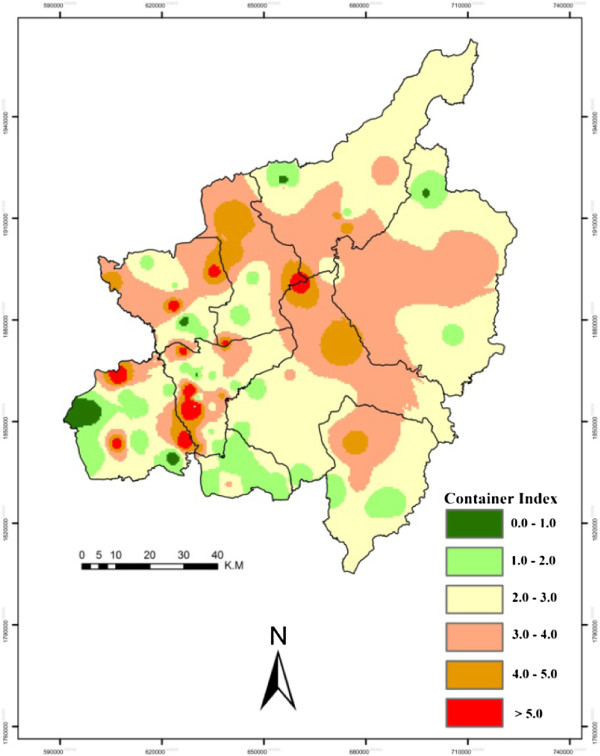
**Province level container index (C.I.).** This map is a visual presentation of C.I., i.e. the percentage of water holding containers infested with larvae, using interpolation techniques on the basis of sampled villages. The legend colors show the levels of C.I. from low (green) to high (red).

**Figure 5:**
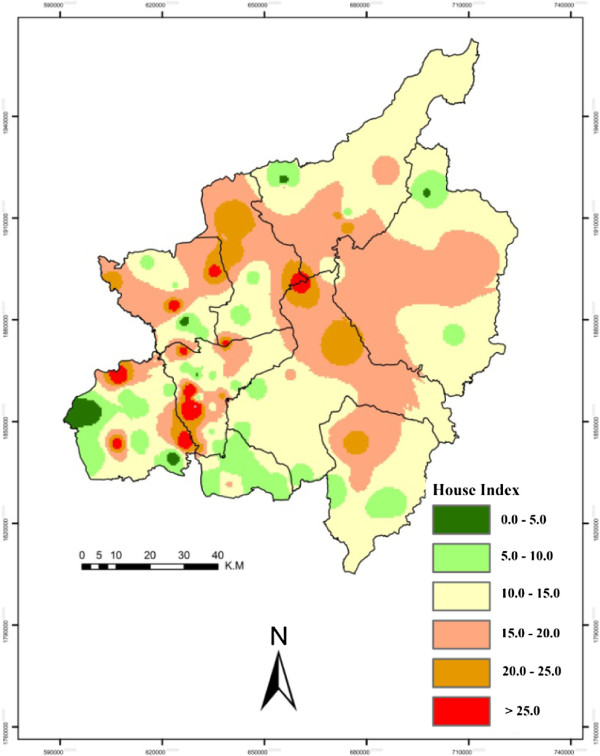
**Province level house index (H.I.).** This map is a visual presentation of H.I., i.e. the percentage of houses infested with larvae, using interpolation techniques on the basis of sampled villages. The legend colors show the levels of H.I. from low (green) to high (red). As per the National Institute of Health (NIH) Thailand, a H.I. of less than 1% is considered to be low risk and greater than 10% is considered to be higher risk.

**Figure 6:**
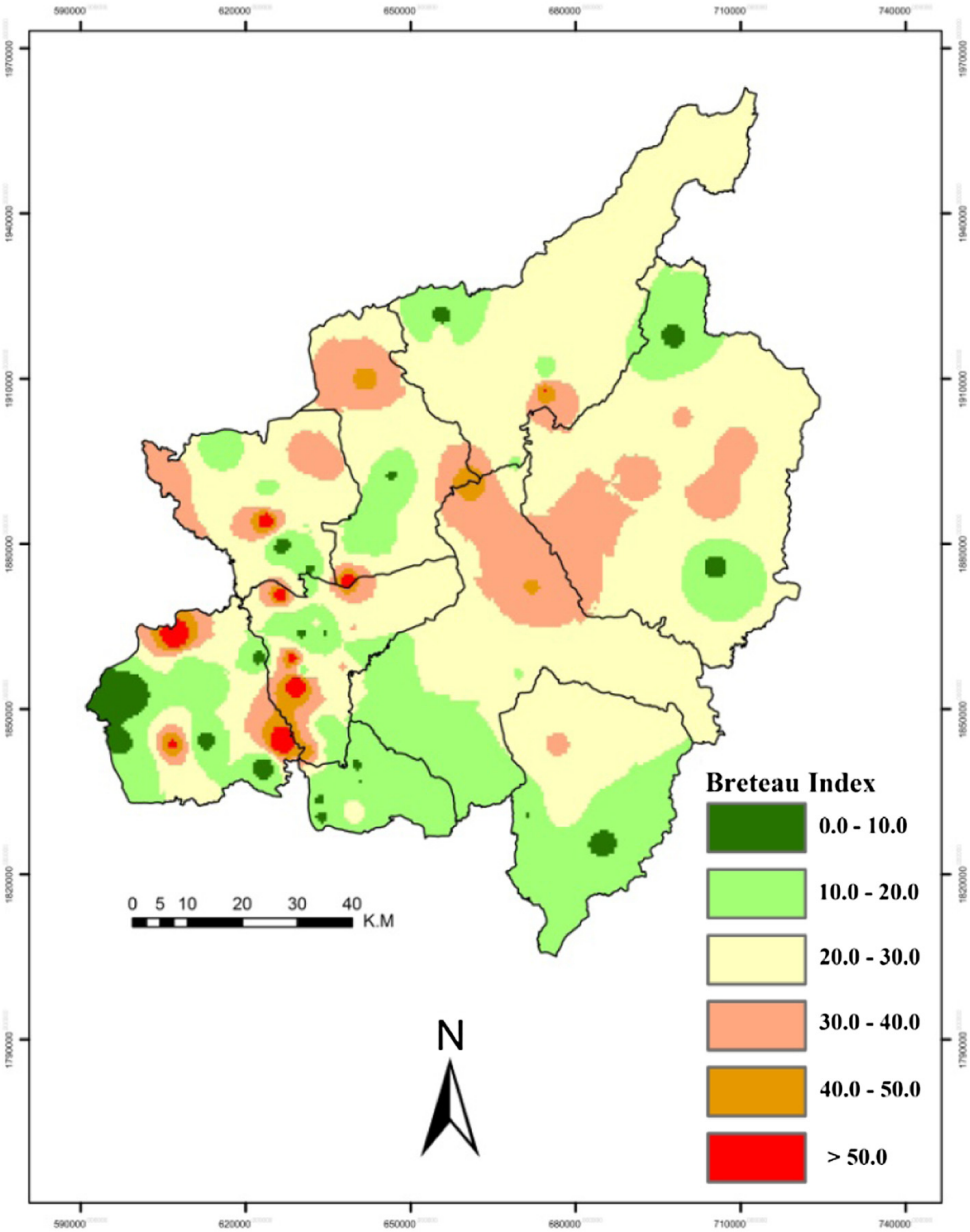
**Province level Breteau index (B.I.).** This map is a visual presentation of B.I., i.e. the number of positive containers per 100 houses inspected, using interpolation techniques on the basis of sampled villages. The legend colors show the levels of B.I. from low (green) to high (red). As per the National Institute of Health (NIH) Thailand, a B.I. of less than 5% is considered to be low risk and greater than 50% is considered to be higher risk.

Dengue indices were observed by trained staff of the Ministry of Public Health (MOPH) in Phitsanulok during the study period. They were well-trained in mosquito species identification and sampling methods. Samples were collected from 92 villages, where dengue cases were reported, and in 2009 sampling were done on three separate occasions in the months of March, May and July. On average 78 houses per village were randomly sampled. Covered indoor and outdoor areas of the selected premises, containing water, also sampled. The different lifestyles of the people and diversity of the surrounding environment were also considered. Out of 92 sampled villages, in 11 villages a total of 29 houses were found locked during revisits; where houses were locked adjacent house was sampled instead, as the neighboring house had almost similar structure and surrounding environment, i.e. land-cover types. Large stagnant ponds, running water and open areas, such as parks and playgrounds were ignored because *Aedes* mosquitoes do not prefer open areas [20,23]. Generally, the peak season for dengue breeding in the study area ranges from April to June and the level of rainfall in the months of April, May and June has a positive effect on dengue larvae; whereas, after July, heavy rains usually start and flush away eggs, larvae and pupae of the dengue vector [24]. May was explored as it was considered to be the peak of the dengue breeding season. Sampled villages were marked with GPS and all the point data was converted into shape files and shifted to GIS layers for spatial epidemiological analysis. Inverse Distance Weighted (IDW) interpolation was performed to indicate the overall situation of C.I. (Figure 4), H.I. (Figure 5) and B.I. (Figure 6) in the study area. ArcGIS 10 ArcMap software was used for GIS analysis, which provides powerful functionality for GIS data and is usually used and recommended by public health professionals [2].

### Spatial and statistical analysis

**Figure 7:**
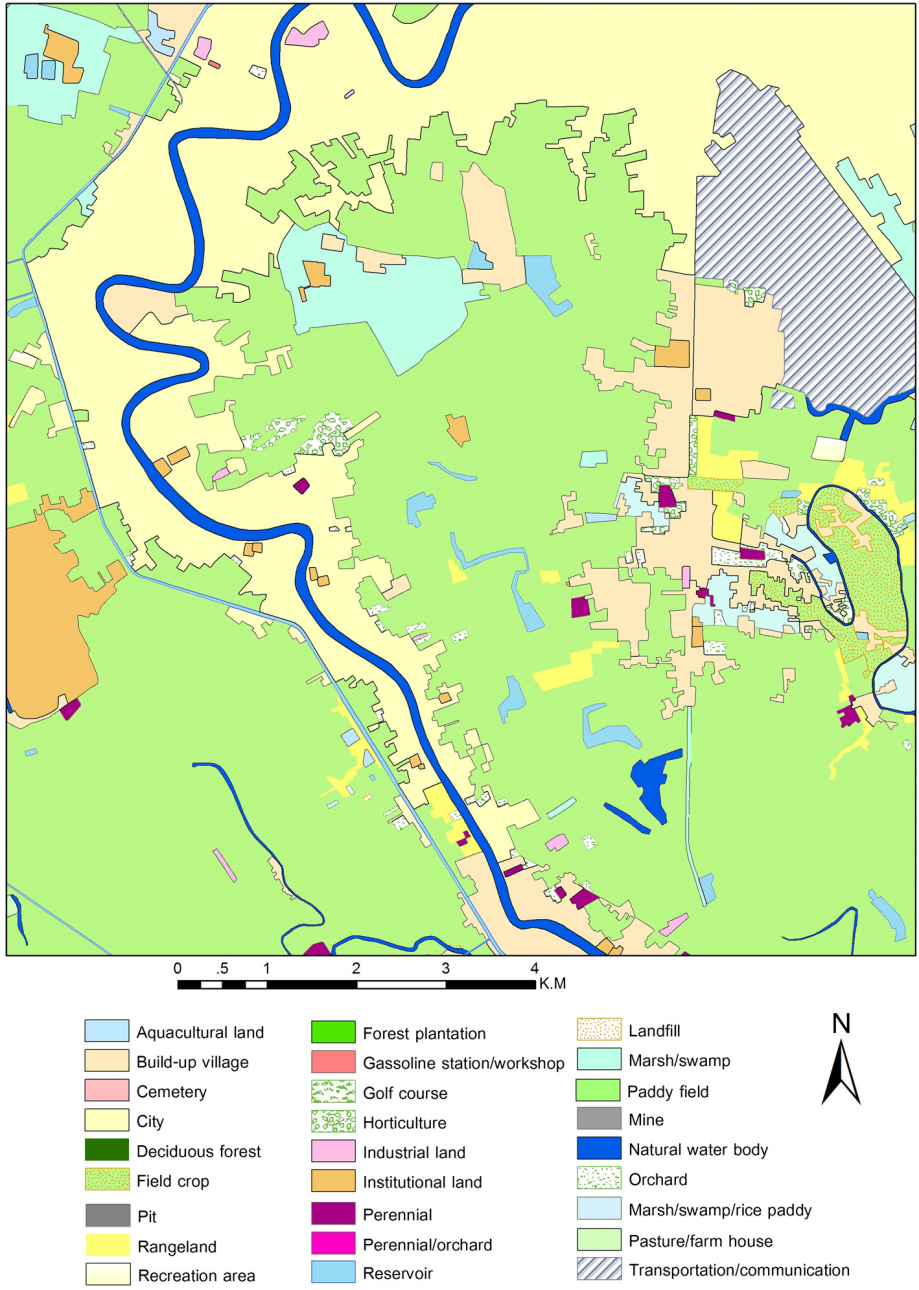
Land-use/land-cover map showing the land-cover types.

Locations sampled were overlaid on a detailed land-cover map to examine the geographically related environmental conditions around villages having different ranges of B.I. Land-cover for 1860 classes was merged into 171 classes following Land Development Department Thailand rules of classification codes, based on similar class structures. The resultant classes were further contracted to 27 classes representing all types of major and mixed classes, as reported in the literature about the relationship of dengue with land-use type [7,11,15] (Figure 7). Successive contractions of land-cover classes were carried out to reduce the redundancy of similar patterns to clearly determine the comprehensive phenomena of vector-borne disease (dengue). Considering the maximum mosquito flight range during its life span around the village, a buffering operation of one-kilometer was performed using GIS software for the 92 villages [15]. This helped to indicate the environmental conditions around the observed locations, i.e. type of trees, crops, fruits and built-up areas, together with all the other factors that play a role in increasing or decreasing dengue indices values. Bivariate Pearson’s correlation analysis was conducted to find out the relationship between mosquito density-related indices and land-use/land-cover classes, which were investigated to explore factors potentially contributing to favorable habitats that facilitate the life-cycle development of dengue-transmitting mosquitoes.

### Factor analysis

Factor analysis, using principal component analysis (PCA) with the varimax rotation method, was performed to explain variance among the land-cover classes to understand the habitat ecology. The factors were analyzed to determine the unique dimensions existing in the data. Prior to conducting the factor analysis procedure, cleaning of data was done, i.e. removal of outlier, verification of data with raw data. Rotation in factor analysis produced results in a more interpretable form and helped to scrutinize the land-cover classes based on their contribution to dengue indices, i.e. B.I., C.I., and H.I. values, either individually or when associated with one or more land-cover classes. Factor analysis is often used to generate hypotheses regarding causal mechanisms and to screen variables for subsequent analysis [25,26].

The factor score is a linear combination of observed variables weighted by eigenvectors. Generation of factor scores is a transformation of observed variables (c_1_ = *β*_11_x_1_ + *β*_12_x_2_ +....... + *β*_1*p*_*x*_*p*_). Where c_1_ is the score of the first component, *β*_1*p*_ is coefficient of regression for observed variable, i.e. its weight and x_*p*_ is observed variable p. The higher weight of the score attached with observed variable (land-use type) represents the higher correlation with independent variable (dengue index). For example, in case of deciduous forest positive loads are given below in equation extracted using rotated component matrix.

$$c_{1}=0.28x_{1}+0.87x_{2}+0.09x_{3}+0.02x_{4}$$

Where, x_1_, x_2_, x_3_ and x_4_ are land types, and from all these land type x_2_ has high loading and x_4_ has less loading. Land-use type x_2_ determines more of variance compared to other land-use types.

The factor model is given as:

Y=XB

Where, Y is a matrix of observed variables, X is matrix of scores for factors and B is matrix of eigenvectors (weights).

Twelve factors were identified using PCA having an eigen value equal to or above 1.0 (Additional file 1). These factors were further grouped from rotated matrix having maximum amount of variance (Table 1, Additional file 2). Furthermore, Bivariate Pearson’s analysis was conducted on score matrix calculating variables having unique dimensions (land-use classes) with respect to dengue indices. Strength of correlation is indicated in Table 2. Statistical analyses were conducted in SPSS (version 16.0).

**Table 1:** Factors selected using principal component analysis

**Factor**	**Groups of variables having unique dimensions**
1	Perennial, orchard and rangeland
2	Recreation area and golf course
3	Deciduous forest and mine
4	Reservoir and pasture/farm house
5	City and aquaculture land
6	Institutional land, build-up village and natural water body
7	Field crop and pit
8	Marsh/swap/rice paddy and gasoline station/workshop
9	Transportation/communication, horticulture and landfill
10	Industrial area and forest plantation
11	Perennial/orchard and cemetery
12	Marsh/swamp and paddy field

One group is treated as one variable during processing of data. Factors scores were used to investigate the contribution of each variable to the dengue indices.

**Table 2:** Correlation between group of land-use type and indices for the months of March, May and July and the study period mean values of each respective index

**Indices**	**Month**	**Factor 1**	**Factor 3**	**Factor 8**
C.I.	March	0.28 ns	0.21^*^	-0.15 ns
	May	-0.01 ns	0.07 ns	0.29^***^
	July	-0.07 ns	0.04 ns	0.18 ns
	Mean	0.08 ns	0.11 ns	0.12 ns
H.I.	March	0.21^*^	0.25^**^	-0.15 ns
	May	0.02 ns	0.06 ns	0.33^***^
	July	-0.10 ns	-0.02 ns	0.29^***^
	Mean	0.03 ns	0.08 ns	0.21^*^
B.I.	March	0.28 ns	0.21^*^	-0.14 ns
	May	-0.01 ns	0.03 ns	0.35^***^
	July	-0.06 ns	0.02 ns	0.19 ns
	Mean	0.09 ns	0.09 ns	0.18 ns

*, ** and *** indicate the strength of correlation at p (probability) <= 0.05, 0.01 and 0.001, respectively Factor 1: orchard, perennial and range land; Factor 3: Deciduous forest and mine; Factor 8: gasoline station/workshop, marsh, swamp and paddy fields.

### Spatial analysis using ring maps

**Figure 8:**
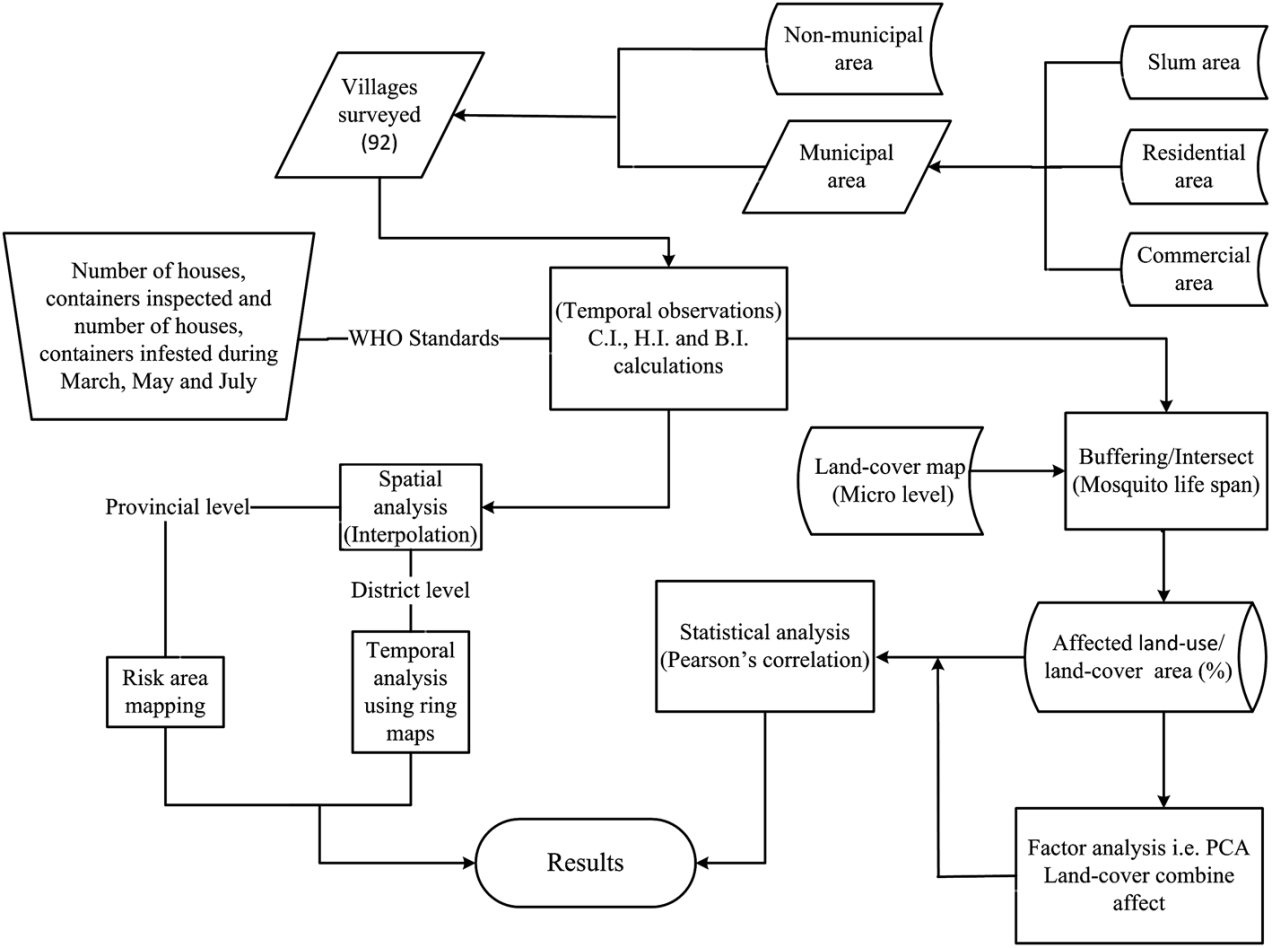
**Average Breteau index (B.I.) throughout the year for Phitsanulok, district-wise (base map) and according to temporal changes (rings).** The base map shows the average B.I. on the basis of interpolation between the villages sampled. Spokes around the base maps indicate village locations, while the inner ring values show average index values of the sampled villages. Extending to the outside from the inner ring, the outer layers represent the index variations during March, May and July, respectively.

**Figure 9:**
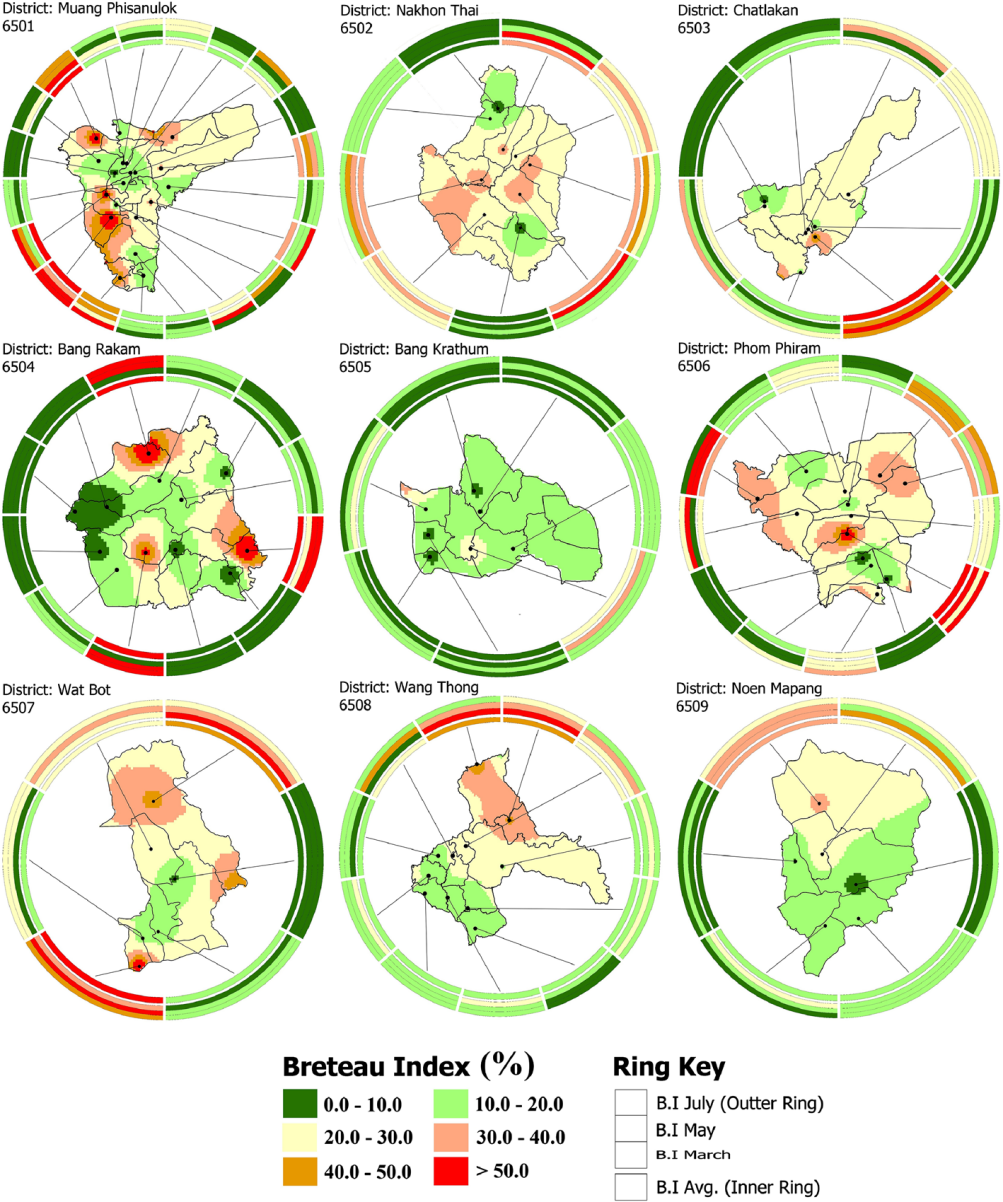
**Relationships among dengue indices throughout the year in Phitsanulok (District-wise).** Average B.I. (base map), average C.I. (inner ring) and average H.I. (outer ring), data was collected with the help of the Ministry of Public Health, Phitsanulok Province. Spokes around the base maps indicate the sampled village locations. The legend colors show the levels of B.I. from low (green) to high (red).

Ring map visualization has been explored as a mean of depicting spatially-referenced, multivariate data in single information graphic. A ring map shows multiple attribute datasets as separate rings of information surrounding a base map of a particular geographic region of interest. Huang et al. [27] used these techniques to map many variables for location specific data and presented a very good picture of temporal data in one graph instead of displaying them on multiple maps. In this study, ring maps were used to visualize spatio-temporal variations of B.I., and to indicate its relationship with respect to C.I. and H.I. Ring mapping provided a direct mean to explore multivariate and temporal data in a single map to evaluate spatio-temporal patterns. Ring maps were generated in Adobe Illustrator with MAPublisher and all attributes information was imported and reclassified according to indices values. In Figure 8, the base map and inner ring represents the average B.I. calculated. Spokes around the base maps indicate village locations surveyed, while the inner ring values show average B.I. of the sampled villages. Extending to the outside from the inner ring, the outer layers represent the index variations during March, May and July, respectively. Every village sampled shows the spatio-temporal changes with respect to B.I. While Figure 9 shows the spatial variation among dengue indices (C.I., H.I. and B.I.) of each surveyed village.

**Figure 10:**
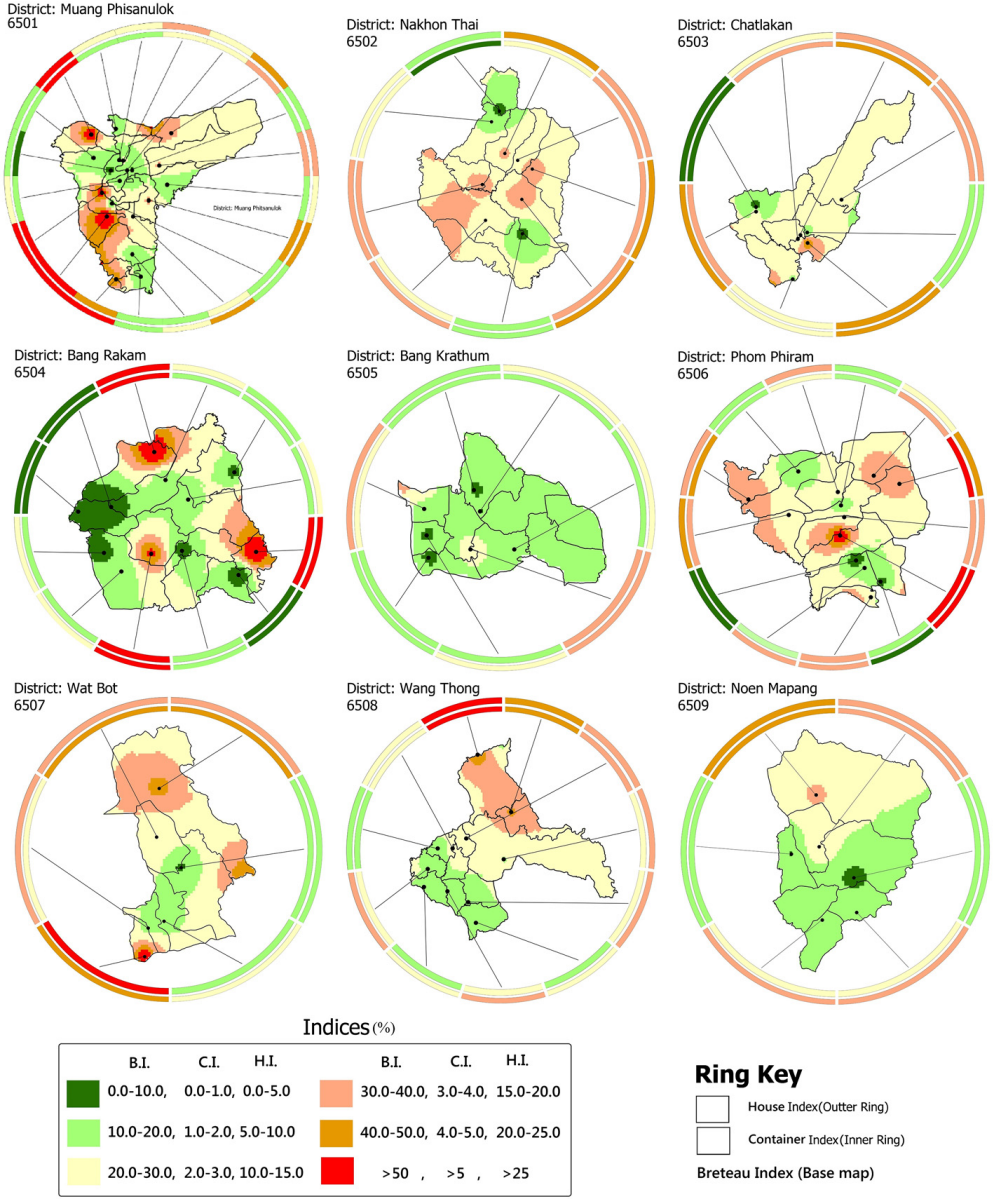
Methodology flow chart.

The overall flow-chart of methodology is shown in Figure 10.

## Results and discussion

### Land-use type

Paddy fields covered the largest area (44%) and gasoline station/workshop occupied the least area (0.01%) in the sampling buffer during the year 2009. Relative land area for deciduous forest, horticulture, institutions, marsh/swamp/rice paddy, and perennial vegetation cover types in the sampling area (buffer) was 4.90, 0.05, 0.79, 1.10, and 2.34%, respectively (Table 3).

**Table 3:** Classified land-use types of study area

**Sr. No.**	**Land-use type**	**M ± SD**	**Sr. No.**	**Land-use type**	**M ± SD**
1	Aquaculture land	0.08±0.40	15	Marsh/swamp/rice paddy	1.07±7.75
2	Build-up village	16.91±14.14	16	Mine	0.07±3.56
3	Cemetery	0.08±0.15	17	Natural water body	2.84±3.56
4	City	6.00±17.95	18	Orchard	2.85±5.12
5	Deciduous forest	4.95±11.46	19	Paddy field	43.94±25.88
6	Field crop	11.58±17.83	20	Pasture/farm house	0.17± 0.65
7	Forest plantation	0.62±5.38	21	Perennial	2.34±5.75
8	Gasoline station/workshop	0.01±0.10	22	Perennial/orchard	0.05±0.28
9	Golf course	0.04±0.40	23	Pit	0.06±0.31
10	Horticulture	0.05±0.20	24	Rangeland	3.98±6.78
11	Industrial land	0.19±0.50	25	Recreation area	0.10±0.66
12	Institutional land	0.79±2.39	26	Reservoir	0.47±1.26
13	Landfill	0.06±0.44	27	Transportation/communication	0.19±0.55
14	Marsh/swamp	0.52±1.87			

M = mean (km^2^); SD = standard deviation.

### Deciduous forest

Previous research has shown that *Ae. albopictus* can have a strong presence in rural areas and forest reserves, inhabiting both macro- and micro-habitats [28,29]. In the current study, population densities of *Ae. aegypti* and *Ae. albopictus* increased as the land under deciduous forest cover increased for the month of March, as there was a significant positive correlation (p ≤ 0.05) observed between house, container and Breteau indices and area under deciduous forest during this month (Table 4). Breeding patterns of *Ae. aegypti* differ according to locality [30] and usually this species prefers to stay within urban environments, but *Ae. albopictus* is a forest mosquito and prefers to breed in tree holes and cavities [29]. Mosquitoes have been observed to be actively present over a wide range within the forest column that could be associated with feeding or ovipositor habits [31]. Some mosquito species are active in the entire vertical forest strata from the ground to the top of the canopy, while others prefer to be either high in the canopy or close to the ground for host-seeking [32,33]. In deciduous forest cover the dominant species of trees and other woody vegetation shed their leaves in cold months and produce new leaves the following spring. The tree species in deciduous forests vary by region, but mostly include oak, beech, elm, walnut, sweet gum, linden, maple, chestnut and hickory, in areas with sufficient rainfall to support tree growth. In forests containing numerous old trees with big trunks, holes and cavities are naturally present and provide resting and nesting sites to various species of birds and other animals [34,35]. In such cavities or holes, the accumulations and retention of rainfall water, together with suitable temperature and humidity ranges, could facilitate the development of a microclimate suitable for the breeding of *Ae. albopictus*. The constant availability of a suitable environment and a variety of hosts in the forest habitat could be another reason for the increased population of *Ae. albopictus* found in containers located within the flight range of this species. These results are in agreement with the observations of Anosike et al. and Derraik et al. [29,36], where forest environments naturally supported the population of the mosquito (vector), but is in disagreement with the findings of Nakhapakorn and Tripathi [15], who observed forests to have a negative effect on dengue incidence. Some researchers have reported the quantitative analysis of mosquito populations in forest habitats [29,36]. The current study was conducted in and around houses, but forest areas lie within the flight range (i.e. within one-kilometer of houses inspected) of the vectors. It was also observed that some hamlets/dwellings were almost surrounded by forest.

**Table 4:** Correlation between land-use type and house, container and Breteau indices for the months of March, May and July and for the study period mean value

**Indices**	**Month**	**Deciduous forest**	**Horticulture land**	**Institutional land**	**Marsh/swamp /rice/paddy**	**Perennial**
C.I.	March	0.211^*^	-0.020 ns	0.107 ns	-0.160 ns	0.300^***^
	May	0.061 ns	-0.039 ns	-0.031 ns	0.295^***^	-0.003 ns
	July	0.037 ns	0.0233 ns	0.095 ns	-0.068 ns	-0.040 ns
	Mean	0.109 ns	0.028 ns	0.074 ns	-0.150 ns	-0.042 ns
H.I.	March	0.254^**^	-0.037 ns	0.267^**^	-0.159 ns	-0.002 ns
	May	0.059 ns	0.224^*^	-0.017 ns	0.337^***^	0.078 ns
	July	-0.031 ns	0.021 ns	0.0109 ns	0.280^***^	-0.047 ns
	Mean	0.076 ns	0.066 ns	0.153 ns	0.208*	0.048 ns
B.I.	March	0.215^*^	-0.003 ns	0.190 ns	-0.143 ns	0.267^**^
	May	0.026 ns	0.110 ns	-0.015 ns	0.349^***^	0.006 ns
	July	0.0190 ns	0.0316 ns	0.114 ns	-0.070 ns	-0.052 ns
	Mean	0.093 ns	0.0227 ns	0.1245 ns	-0.150 ns	-0.021 ns

*, ** and *** indicate the strength of correlation at p (probability) < = 0.05, 0.01 and 0.001, respectively.

### Horticulture

The horticulture land-use class includes the cultivation of bamboo, truck crops, floriculture, vine, pepper, strawberry, passion fruit, raspberry, herbs, grasses, etc, and is characterized by the presence of regularly applied irrigation to maintain landscape vigor and associated production of fruits and flowers. Male mosquitoes feed usually on floral nectar, sucking sugar-containing syrup as their staple food [37-39]. Since males don’t bear the burden of producing young-ones, they avoid human biting and mostly feed on floral nectars [17]. This nutritious syrup is found in floral nectars, at the base of leaves and soft stems, and even less frequently, on fruit [40,41]. This is also observed as exuding from damaged plants as honeydews [42-44]. Mosquitoes also feed directly from plants by piercing undamaged tissue [45,46]. Mostly the female *Aedes* mosquito feeds on human blood. This is because these need the protein found in blood to produce eggs [17]. Bamboo, as a horticultural plant could provide a suitable breeding habitat for mosquitoes, especially *Ae. albopictus*[47,48]. Hence, horticultural land serves as one of the major breeding places for mosquitoes. In the present study, the number of houses infected with dengue significantly increased with increasing horticultural land (p = 0.05) and a positive relationship was observed between H.I. and the area under horticultural land-use for the month of May (Table 4). Availability of moisture due to irrigation, or abrupt increases in rainfall, in the month of May might have supported the development of a suitable breeding environment for *Ae. albopictus* in horticultural zones and possible propagation of adult mosquitoes to containers in houses within their flight range. Increased mosquito populations in or around horticultural land during the months of May and June due to increased rain showers was also observed [49]. Furthermore, the results are in agreement with the findings of Vanwambeke et al. [11], who observed a positive relationship between the population of *Ae. albopictus* and area of horticultural land-cover.

### Integrated effect of land-use type

The relationship of groups of land-use types with house, container and Breteau indices with their mean values is presented in Table 1. As a result of factor analysis, land-cover types including factor 1 (orchard, perennial and rangeland) have a significant correlation (p = 0.05) with the number of houses containing infected containers (H.I.) during the month of March. The obvious reason for this significant correlation was high vector population during this period. Mosquitoes feed on floral nectar, fruit juices and liquids exuded from plants, and nectar sugar is burned as fuel for flight and is replenished on a daily basis, whereas blood protein is needed for egg production, so is imbibed less frequently [57]. Plant axils, as in bananas, are favorable for dengue vector, but these feed mainly on nectar from wildflowers. Also, the peak mosquito spawning season coincides with the peak blossoming of fruit trees [63]. Rangeland and perennials consist of grass, shrub, bamboo, etc; where the bamboo can contribute to the availability of favorable habitats for dengue vectors [48]. Factor 3 (deciduous forest and mines) had a combined significant correlation (p ≤ 0.05) with house, container and Breteau indices for the month of March. As already explained, forest environment provides feeding and breeding habitat to *Ae. albopictus* dengue mosquito. Similarly, Factor 8 (gasoline station/workshop, marsh, swamp and paddy fields) was significantly correlated (p = 0.001) with the H.I. for the months of May and July with an average value (p = 0.05) for this study period, and with B.I. (p = 0.001) for the month of May. The commonality among such land-use types could be the presence of water either in fields, such as rice paddies and swamps, or in containers, like tyres from nearby gasoline stations [64]. Studies by various investigators [57,59,64] have shown the presence of dengue vectors at gasoline station/workshops, marsh and paddy fields; however, none of the studies reported indices.

Type of land-use in most cases remained the same within the period from March to July; however, it was noted that the habitat characteristics determined by the stage of vegetation (vegetative, flowering), management practices (irrigation, sowing, harvesting) and rainfall pattern varied across the said duration. Some flowering plants had already reached post flowering stages in March and thereby the same vegetation, only newly seeded, was at its full blooming stage during the month of May and became an attraction for and served as a supporting habitat to many insects including mosquitoes for nectar feeding during this month. Depending on the differences in number of houses inspected (H.I.) and number of containers infested (C.I.), behavior indices varied from each other. This happened when the number of inspected houses increased or decreased with only a slight change in the number of infested containers. In May, the number of houses infested increased but the number of infested containers within a house remained the same, and so, in some cases this would not result in a respective change in C.I. and B.I.

### Institutional land

With local increases in human population, new schools and other institutional facilities are continuing to be developed. Institutional land-cover includes facilities for education, religion, healthcare, military, etc. All buildings, including grounds, parking lots and other auxiliary land-uses, are included within this land-use type. In the present study, greater institutional land area resulted in enhanced dengue mosquito density in houses within one-kilometer radius and a strong correlation (p = 0.01) existed between the area under institutions and the number of houses with infected containers (Table 4). *Ae. aegypti* is well-adapted to populated environments and typically breeds in clean, stagnant water (rain water) in proximity to human dwellings [23,50,51]. Water containers, such as roof gutters, catch basins, rain barrels, swimming pools, wading pools, cesspools, septic tanks, tyres, paint buckets, tin cans, yard debris, coconut shells, bottles and rubbish collecting units are usually present in or around institutional buildings in urban areas and could serve as breeding sites for dengue vectors [52]. In people attending institutions, dengue infections have been widely reported. Most of the virus transmission occurred in people within these institutions, and such places are likely being the most appropriate habitat for mosquito breeding and dengue epidemic as well [53,54]. Previous reports also indicate that most of the surveyed institutions were found positive for *Ae. Aegypti*, and therefore special attention is required to such high potential breeding sites [55,56].

### Marsh/swamp/rice paddy

Marsh/swamp is an open place which holds standing water. Paddy fields are uncultivated areas having standing water, which have been prepared for rice crop to be sown in near future. Rice paddy fields have almost identical characteristics, but possess growing rice crop. All three were considered to be a single land-cover type. Marshes feature an abundance of reeds, rushes and cattails, and are generally treeless and open spaces, whereas swamps are either completely or partially covered with trees and shrubs. A wide variety of birds, such as ducks, swans, terns, geese, herons and songbirds live in marshes and swamps. Rice paddy fields contain rice crops for about four months, two times a year in Thailand (total of 8 months per year). Marsh, swamp and rice paddy are all characterized by standing water from 2.5 cm to 30 cm in depth, and also serve as catchments for the rain water. Mosquitoes commonly breed in areas with standing water or temporary pools like salt marshes, swamps and areas flooded by snow melts and spring rains [57,58]. In the study area, the areas located in proximity to land containing marshes, swamps and paddy fields possess increased dengue vector population. This increase was highly significant, as indicated by correlations between land covered with marsh, swamp and rice paddy and B.I. (p = 0.001) for the month of May and also with the average H.I. (p = 0.05) for all three months (Table 4). Although major vector species of the genus *Aedes* (*Ae. aegypti* and *Ae. albopictus*) are known as container-breeding species and prefer small volumes of water in artificial environments (urban), these species can also be expected in natural situations. They breed in water collected in dead leaves, tree holes and rock pools observed in marshes, swamps and rice paddies, and can also feed on birds living in marshes and swamps [59]. They might also have access to containers from houses in the vicinity. Marshes, swamps and rice paddies have been reported as a source of vectors for many other diseases, but recently it has been observed that the still water at such places is a suitable habitat for dengue vectors. It has been recommended, as a precautionary measure, to stay away from such habitats [60,61]. Also, many farmers build temporary houses in rice paddy fields and live there throughout the growing period until harvest. They store water in earthenware containers which serve as potential habitats for dengue vectors.

### Perennial

Perennial vegetation in this study includes para rubber, coconut, oil palm, teak, magosa, mangrove, coffee and tea plantations, all of which can serve as breeding sites for *Ae. Albopictus*. These can all serve as substitutes for the regular components of the tree hole fauna [28,29] and natural containers used for development of its larvae and pupae, as these have been found in tree holes and coconut shells [62]. In present study, a greater area of perennial vegetation is found to have a positive correlation with the density of *Ae. aegypti* and *Ae. albopictus* in containers placed in houses within the flight range of adult mosquitoes. Particularly, a significant and positive correlation (p ≤ 0.05) was observed between the number of containers infected with dengue and B.I. for the month of March (Table 4). A possible reason could be the presence of constant favorable habitat due to moderate microclimate and breeding sites (tree trunks, leaves and tree holes) that facilitated the growth of *Ae albopictus*. This is in agreement with Anosike et al. [29] and Mangudo et al. [62] who reported the favorability of woody tree vegetation environments for the growth of *Ae. albopictus*.

### Spatial temporal analysis of dengue indices

Spatial and temporal analyses were performed at provincial and district levels. Provincial level results were extracted and are shown in Figures 4, 5 and 6, while the district level temporal and spatial changes can be seen in Figures 9 and 10 in ring maps.

Out of the B.I. sampled villages in districts 6501 to 6509, 0, 0, 13, 25, 0, 8, 0, 0, and 0% had optimal B.I.; and 15, 0, 12, 25, 0, 8, 17, 0, and 0% had emergency B.I., respectively (Figure 9, Table 5). Out of the total sampled villages in districts 6501 to 6509, 5, 11, 0, 0, 0, 8, 0, 0, and 0% had optimal H.I., and 70, 67, 87, 67, 87, 67, 86, 69, 83, 91 and 50% had emergency, H.I., respectively (Figure 9, Table 6). According to the risk levels set by the National Institute of Health (NIH), Thailand, optimal control is a H.I. < 1% and emergency levels of infestation are indicated by a H.I. of 10% or greater. Similarly, a B.I. < 5% is consider to be an optimal index and > 50% is consider as high risk [24]. C.I. only provides information on the proportion of water-holding containers that are positive. The B.I. and H.I. are commonly used for the determination of priority (risk) areas for control measures. Figure 8 shows the detailed results generated using ring maps for each district of Phitsanulok Province in terms of B.I. Figure 9 shows the relationship between average B.I., C.I. and H.I. Common breeding habitats found in study area are shown in Additional file 3.

**Table 5:** Number of villages with optimal, moderate, alarm and emergency Breteau index (B.I.)

**District no.**	**Sample villages**	**Optimal index**	**Moderate index**	**Alarm index**	**Emergency index**
6501	20	0	3	14	3
6502	9	0	2	7	0
6503	8	1	0	6	1
6504	12	3	3	3	3
6505	7	0	4	3	0
6506	13	1	1	10	1
6507	6	0	1	4	1
6508	11	0	0	11	0
6509	6	0	2	4	0

According to the risk levels set by National Institute of Health (NIH), Thailand optimal control is a B.I. < 5% and emergency levels of infestation are indicated by a B.I. of 50% or greater. Further as per Ministry of Public Health Thailand (MOPH), Phitsanulok two more groups were categorized, i.e. B.I. between 5% and 10% as moderate index and B.I. between 20 to 50% as alarm index.

**Table 6:** Number of villages with optimal, moderate, alarm and emergency house index (H.I.)

**District no.**	**Sample villages**	**Optimal index**	**Moderate index**	**Alarm index**	**Emergency index**
6501	20	1	0	5	14
6502	9	1	1	1	6
6503	8	0	0	1	7
6504	12	0	0	4	8
6505	7	0	0	1	6
6506	13	1	1	2	9
6507	6	0	0	1	5
6508	11	0	0	1	10
6509	6	0	1	2	3

According to the risk levels set by National Institute of Health (NIH), Thailand optimal control is a H.I. < 1% and emergency levels of infestation are indicated by a H.I. of 10% or greater. Further as per Ministry of Public Health Thailand (MOPH), Phitsanulok two more groups were categorized, i.e. H.I. between 1% and 5% as moderate index and H.I. between 5 to 10% as alarm index.

Heavy rains and floods may increase the risk of infectious diseases, such as typhoid fever, cholera, leptospirosis and hepatitis A, and vector-borne diseases like dengue, malaria, yellow fever, and West Nile fever [65]. Often flood generate favourable breeding habitats for *Aedes* and thus may result in increase of vector population in flooded area. This may lead to potential risk of dengue outbreak following a flood. But there are no immediate effects as floods interrupts vector-borne disease cycle as it alters vector breeding habitats and in result may cause reduction in dengue for the time [66]. Flood may change land-use and so can directly affect the habitat of dengue due to water availability, such as increase in marsh/swamp areas, increase in water fill in used tyres around workshops, etc. can result in more breeding places. Hence, vector growth can re-emerge within 3 to 4 weeks following the flood. But good control programs like cleaning operations, disposing of unused water containers including old tyres, tin-cans, broken jars and plastic bottles, etc. can lead to better control on vector population. As per report of MOPH Thailand, the number of dengue fever cases fell by 36% in Thailand after 2011 flood that was the worst flood during five decades. MOPH, Thailand vector-borne diseases took serious efforts to reduce breeding habitats and so it may be the reason of less infection in next year.

### Accuracy assessment of statistical methods

**Figure 11:**
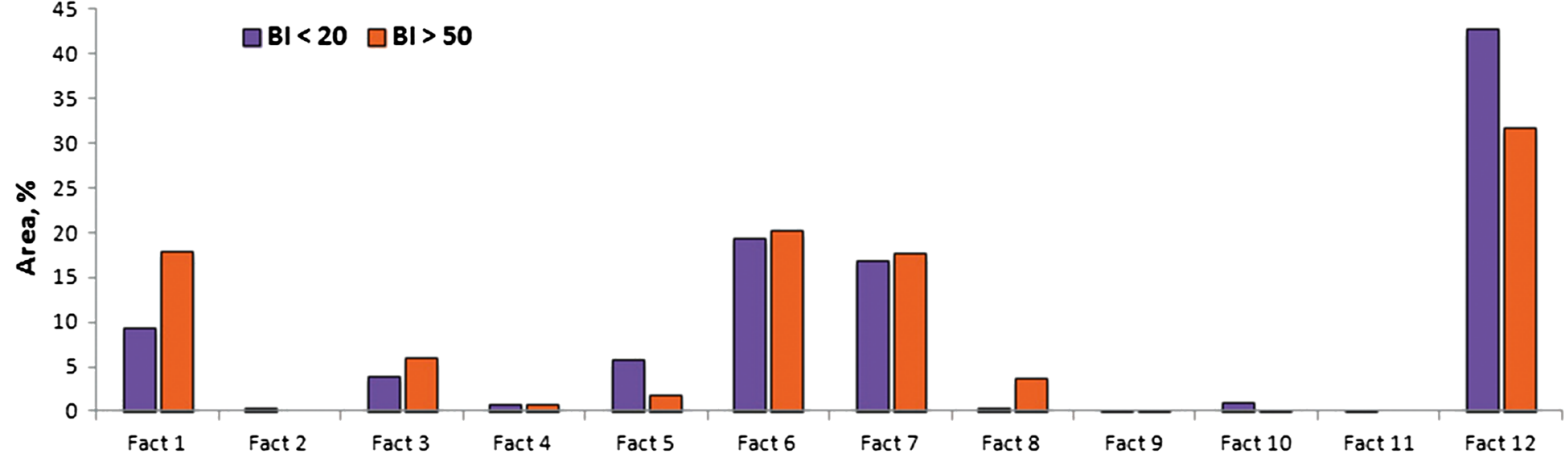
**Relationship between low and high Breteau index (B.I.) having different land-cover and land-use.** Twelve representative factors were extracted on the basis of factor analysis. Equal/approximately equal percentage of area showed that there was no difference between land-cover/land-use having a B.I. of less than 20 and a B.I. of greater than 50. Difference in land-cover and land-use area (percentage) represents significance difference in B.I.

Figure 11 is the graphical representation of verification between spatial and statistical results. A similar pattern was observed for the areas of high and low B.I. However, out of a total of twelve factors seven showed no significant difference for B.I., indicating the complexity of dengue habitats with land-cover types. Thus, high B.I. was found to be associated with a variety of land-cover types including perennial, orchard, rangeland, deciduous forest and mixed marsh/swamp/rice paddy land-cover with nearby gasoline stations/workshops. Factors associated with low B.I. mainly include the land-cover types, such as aquaculture and non residential urban (city). Factors having land-use types, such as mixed marsh/swamp and rice paddy fields, have been found to disagree with the statistical results. This could be due to there being very few places that have workshops nearby, so making them unsuitable as habitats for dengue vectors.

### Limitations and future recommendations

In order to explore the natural/pragmatic phenomena for expected dengue risk, randomly selected houses having water containers of different sizes, shapes and numbers were observed. One of the limitations of this study is that the containers vary from house to house with respect to their size, shape and more importantly their number. To combat this complexity related to number of containers a ratio between infested and inspected containers was adopted. It might be the reason for variation of B.I., H.I. and C.I. For example, the relationship between dengue indices, as shown through ring mapping (Figure 9), indicates that many villages contain higher H.I. as compared to C.I. and B.I. This indicates that increases or decreases in the number of inspected houses does not always bring a change in the number of infested containers and consequently in the B.I. at the same rate. Hence, behavior of H.I., C.I. and B.I. can be different depending on the existing scenario. Owing to lack of resources data of 2009 survey was investigated in the present study. Furthermore, a weak correlation with a single factor, i.e. land-cover/land-use can be expected owing to the multi-factorial complex phenomena involved in determining the scenario. Availability of larval data might be beneficial in predicting more precisely the suitable artificial and natural containers in study area.

The unpredictability and complexity of infectious disease is still a challenge for health managers and decision makers, because several factors contribute the outbreak. Climatic, economic, biological, ecological and socio-economic factors are certainly among the other factors which compile suitable/unsuitable environments for dengue vector propagation, and by integrating these, a useful model can be developed. The resultant multi-factorial relationship with dengue indices and land-cover types may help elucidate the complex phenomenon of dengue outbreak more precisely. This type of geo-spatial approach can help public health department to take timely dengue control measures to thwart disease outbreak.

## Conclusions

In the present study, settlements with a greater number of gasoline stations/workshops and institutional areas had higher B.I. values, suggesting that these factors significantly contribute to the favorable dengue vector habitats. Dwellings surrounded by marsh/swamp located near paddy fields proved suitable for the growth of dengue vector populations, especially during the rainy season. The strong relationship between deciduous forest, horticulture and perennial areas, and dengue indices indicates that such vegetation is potential habitat for *Aedes*. These findings show that the presented micro-level datasets created from analysis using statistical methods and spatial tools are beneficial in predicting dengue habitat to facilitate early public health responses. These findings along with reported climatic and demographic factors have special significance in minimizing or curbing the potential risk of dengue outbreak morbidity and mortality. However, these indices weakly predict dengue cases, but can present a fairly close scenario of suitability of dengue habitat. A clear understanding of the procedures for spatial factor assessment and temporal analysis facilitates identification and display of potential predictors for the incidence of dengue in the near future and the risk associated with this disease, and will provide a novel way to start investigations. In conclusion, the use of integrative technologies can enable the healthcare mangers to take early decisions to prevent the spread of dengue outbreak.

## Competing interests

The authors declare that they have no competing interests.

## Author’s contributions

MSS, TT and PK participated in data collection with the collaboration of Ministry of Public Health, Thailand team. MSS carried out the analysis and drafted the initial manuscript while Dr. NKT, Dr. TT and Dr. MS designed the conceptual approach and also assisted in the analysis of data. All authors jointly contributed to the revision and approved the final manuscript.

## Pre-publication history

The pre-publication history for this paper can be accessed here:

http://www.biomedcentral.com/1471-2458/12/853/prepub

## Supplementary Material

Additional file 1 Rotated component matrix obtained from factor analysis. Click here for file

Additional file 2 Factors integration on the basis of rotation matrix. Click here for file

Additional file 3 Common breeding habitats found in study area. Click here for file
