# Supplementary material for: Analyzing the spatio-temporal relationship between dengue vector larval density and land-use using factor analysis and spatial ring mapping
Source: BMC Public Health. 2012 Oct 9;12:853. doi: 10.1186/1471-2458-12-853 (PMC3598814; doi:10.1186/1471-2458-12-853)
Supplement: Additional file 1 — Rotated component matrix obtained from factor analysis. [file 1471-2458-12-853-S1.pdf]

Additional file 1 --- Rotated component matrix obtained from factor analysis

S. No	Land-use type	Factor 1	Factor 2	Factor 3	Factor 4	Factor 5	Factor 6	Factor 7	Factor 8	Factor 9	Factor 10	Factor 11	Factor 12
1	Aquaculture land	-0.04	-0.02	-0.06	-0.05	**0.79**	0.06	-0.06	0.00	-0.08	-0.13	-0.02	-0.07
2	Build-up village	-0.20	0.11	-0.18	0.05	-0.28	**0.62**	-0.07	0.03	0.38	0.10	-0.06	-0.16
3	Cemetery	-0.23	-0.04	0.05	0.12	-0.03	-0.13	0.22	0.02	-0.03	-0.21	**0.65**	-0.16
4	City	-0.10	-0.03	-0.03	-0.01	**0.84**	-0.17	0.02	-0.05	0.07	0.27	-0.05	0.04
5	Deciduous forest	0.28	-0.04	**0.87**	-0.01	-0.06	-0.10	0.09	-0.02	-0.02	-0.09	0.02	-0.13
6	Field crop	0.28	-0.10	0.03	0.14	-0.15	-0.28	**0.70**	-0.06	-0.16	-0.22	-0.06	-0.22
7	Forest plantation	0.06	-0.10	-0.06	-0.02	-0.17	-0.25	-0.45	-0.09	-0.29	**0.20**	-0.18	-0.17
8	Gasoline station/workshop	-0.01	0.00	-0.01	0.17	-0.04	-0.05	-0.04	**0.79**	-0.04	0.14	-0.03	0.19
9	Golf course	-0.02	**0.89**	-0.01	-0.02	-0.03	-0.03	-0.01	-0.02	-0.02	-0.01	0.00	0.03
10	Horticulture	0.26	-0.04	-0.01	-0.13	-0.08	0.07	-0.12	0.00	**0.68**	-0.05	0.13	-0.02
11	Industrial land	0.00	-0.03	0.00	0.04	0.06	-0.02	0.04	0.10	0.10	**0.81**	-0.02	-0.10
12	Institutional land	-0.09	-0.12	-0.05	0.07	-0.08	**0.63**	0.01	-0.05	0.02	-0.08	-0.12	0.04
13	Landfill	-0.20	-0.01	-0.11	0.03	-0.15	-0.49	0.11	-0.01	**0.19**	0.03	-0.16	-0.18
14	Marsh/swamp	-0.05	-0.01	-0.04	0.05	-0.02	0.02	0.01	0.09	0.09	-0.08	-0.09	**0.81**
15	Marsh/swamp/rice paddy	-0.03	-0.03	-0.03	-0.06	-0.01	0.02	-0.01	**0.86**	-0.03	-0.03	0.00	-0.07
16	Mine	-0.11	-0.01	**0.92**	0.00	-0.04	-0.01	-0.06	-0.03	-0.04	0.05	-0.01	0.06
17	Natural water body	-0.13	0.41	-0.10	-0.08	0.06	**0.56**	0.06	0.00	-0.06	0.36	-0.02	-0.30
18	Orchard	**0.70**	-0.03	-0.02	-0.11	-0.14	-0.02	0.03	-0.08	-0.04	0.13	0.24	0.28
19	Paddy field	-0.56	0.00	-0.29	-0.17	-0.23	-0.04	-0.43	-0.16	-0.10	-0.08	0.12	**0.32**
20	Pasture/farm house	0.14	-0.02	-0.03	**0.85**	-0.02	-0.09	0.01	-0.09	-0.08	-0.14	0.27	-0.11
21	Perennial	**0.74**	-0.04	0.02	0.03	-0.06	-0.06	0.09	-0.02	-0.01	0.01	-0.04	-0.04
22	Perennial/orchard	0.22	-0.01	-0.04	0.02	-0.04	0.02	-0.16	-0.04	0.04	0.11	**0.79**	0.02
23	Pit	0.04	-0.04	-0.05	-0.13	-0.06	-0.01	**0.70**	-0.09	-0.18	0.30	-0.03	0.09
24	Rangeland	**0.60**	0.05	0.09	0.13	0.00	-0.09	-0.03	-0.01	0.06	-0.36	-0.01	-0.24
25	Recreation area	0.01	**0.90**	-0.02	0.00	-0.03	0.01	-0.04	-0.01	-0.02	-0.05	-0.03	-0.03
26	Reservoir	-0.05	-0.01	0.03	**0.86**	-0.06	0.16	-0.02	0.19	0.01	0.14	-0.13	0.17
27	Transportation/communication	-0.18	-0.04	-0.03	0.05	0.05	-0.07	-0.04	-0.08	**0.78**	0.16	-0.12	0.12

These numbers in the matrix are the proportion of each variable's variance that can be explained by the factors. The values given in red have high loading. For example, orchard, perennial and rangeland have high loading value in column one, and all are considered in factor 1.
