# Supplementary material for: Analyzing the spatio-temporal relationship between dengue vector larval density and land-use using factor analysis and spatial ring mapping
Source: BMC Public Health. 2012 Oct 9;12:853. doi: 10.1186/1471-2458-12-853 (PMC3598814; doi:10.1186/1471-2458-12-853)
Supplement: Additional file 2 — Factors integration on the basis of rotation matrix. [file 1471-2458-12-853-S2.pdf]

Factors integration on the basis of rotation matrix

Factors	Variables	Factor 1	Factor 2	Factor 3	Factor 4	Factor 5	Factor 6	Factor 7	Factor 8	Factor 9	Factor 10	Factor 11	Factor 12
1	Perennial, Orchard, Rangeland	0.74 0.70 0.60											
2	Recreation area, Golf course		0.90 0.89										
3	Mine, Deciduous forest			0.92 0.87									
4	Reservoir, Pasture/farm house				0.86 0.85								
5	City, Aquaculture land					0.84 0.79							
6	Institutional land, Build-up village, Natural water body						0.63 0.62 0.56						
7	Field crop, Pit							0.70 0.70					
8	Marsh/swap/rice paddy, Gasoline station/workshop								0.86 0.79				
9	Transportation/communication, Horticulture, Landfill									0.78 0.68 0.19			
10	Industrial area, Forest plantation										0.81 0.20		
11	Perennial/orchard, Cemetery											0.79 0.65	
12	Marsh/swamp, paddy field												0.81 0.32

All factors were grouped on the basis of loading values. Factor 1 represents (perennial, orchard and rangeland), factor 2 represents (recreation area and golf course) and so on.
