# Supplementary figures and images for: Analyzing the spatio-temporal relationship between dengue vector larval density and land-use using factor analysis and spatial ring mapping
Source: BMC Public Health. 2012 Oct 9;12:853. doi: 10.1186/1471-2458-12-853 (PMC3598814; doi:10.1186/1471-2458-12-853)

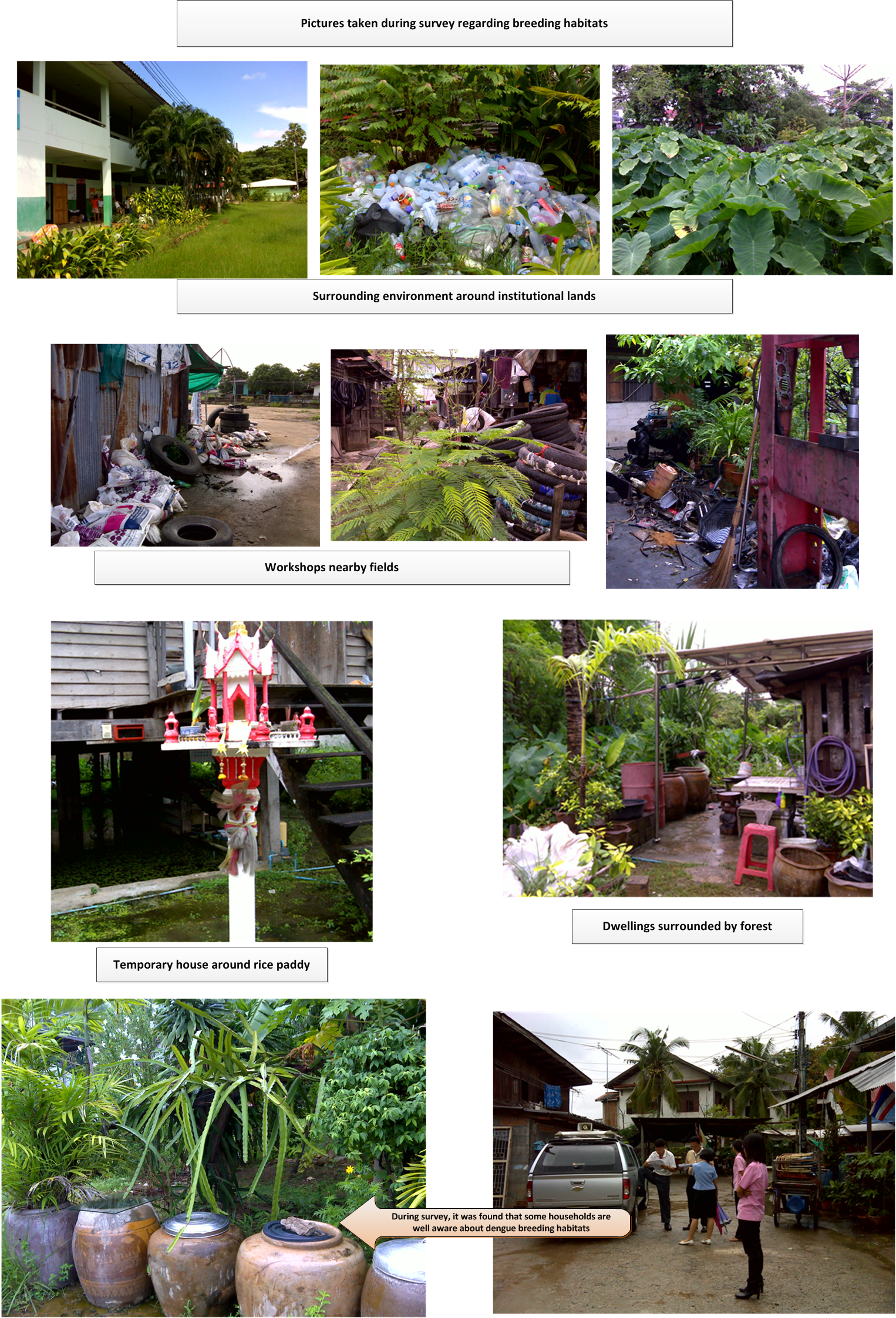

Supplement: Additional file 3 — Common breeding habitats found in study area. [file 1471-2458-12-853-S3.tiff]
